# Estrogen induces shift in abundances of specific groups of the coral microbiome

**DOI:** 10.1038/s41598-021-82387-x

**Published:** 2021-02-02

**Authors:** Caren L. S. Vilela, Helena D. M. Villela, Gustavo A. S. Duarte, Erika P. Santoro, Caio T. C. C. Rachid, Raquel S. Peixoto

**Affiliations:** 1 Department of General Microbiology, Paulo de Goes Institute of Microbiology, Federal University of Rio de Janeiro, Rio de Janeiro, Brazil; 2 Red Sea Research Center (RSRC), Division of Biological and Environmental Science and Engineering (BESE), King Abdullah University of Science and Technology (KAUST), Thuval, Saudi Arabia

**Keywords:** Microbial ecology, Water microbiology

## Abstract

Synthetic estrogens such as ethinylestradiol (EE2) are persistent micropollutants that are not effectively removed from wastewater by conventional treatments. These contaminants are released into waterbodies, where they disrupt endocrine systems of organisms and cause harmful effects such as feminization, infertility, reproduction problems and genital malformations. The consequences of this pollution for key marine ecosystems such as coral reefs and their associated microbiomes are underexplored. We evaluated the effects of EE2 concentrations of 100 ng L^−1^ and 100 µg L^−1^ on the coral metaorganism *Mussismilia harttii.* The results indicated no effects on visible bleaching or *F*_v_/*F*_m_ ratios in the corals during a 17-day microcosm experiment. However, next-generation sequencing of 16S rDNA revealed a statistically significant effect of high EE2 concentrations on OTU richness, and shifts in specific microbial groups after treatments with or without EE2. These groups might be bioindicators of early shifts in the metaorganism composition caused by EE2 contamination.

## Introduction

Estrogenic hormones are part of a group of endocrine-disrupting compounds (EDCs) that can alter the endocrine system of animals^1–3^. 17α-ethinylestradiol (EE2) is a synthetic hormone used in birth-control pills and in hormone-replacement drugs in the menopausal period, or to treat other hormonal deficiencies^4^. EE2 is derived from the main endogenous human estrogen, estradiol (E2), but is 10 to 50 times more powerful^5,6^. Its longer half-life makes EE2 more available for bioaccumulation by other animals^7–10^. This compound is released into the environment through human excreta, and is persistent^4,11^.

Studies have shown that EE2 can cause estrogenic effects in very low concentrations (1 to 5 ng L^−1^), with a predicted no-effect concentration of 0.35 ng L^−1^^12^. However, EE2 concentrations of 0.001 to 0.042 µg L^−1^^13^ or higher, of 0.831 μg L^−1^^14^ have been reported in some wastewater-treatment plants and their receiving water bodies, and may come into direct contact with wildlife^2,7,15–17^. The presence of estrogens in water bodies is responsible for female infertility, feminization of male fish, and sexual dysfunction of many aquatic species^2,3,18–20^. These compounds also affect the microbiome associated with different organisms, such as the insect *Megaselia scalaris*, which had its development impacted by a hormone diet (including EE2), with a significant effect on microbial groups compared with control samples and the appearance of *Mycobacterium, Sphingobacterium, Nocardioides, Acinetobacter* and other genera^21^. Mosquitoes exposed to hormone treatment in environmentally important concentrations showed a significant difference in their microbiome compared to untreated samples^22,23^. In one study, *Microbacterium laevaniformans* was the most abundant species in hormone-treated samples, whereas *Wolbachia pipientis* was most abundant in the control^23^. Continuous use of human oral contraceptive pills was found to alter the normal vaginal microbiome and increase yeast colonization^24^. These examples from humans and insects are used as references due to the lack of information regarding the effects of EE2 on the coral-associated microbiome. The investigation of the impacts of EE2 on other animal models and their associated microbiomes will add to the current body of information regarding the importance of this pollutant in potentially symbiotic microbial populations.

The effects of estrogens on corals have been examined in a few studies focusing on their metabolism, reproduction and physiology^25–29^. Sex steroids are biologically active in invertebrates, including corals, although their mechanisms of action remain unclear^26,30^. It is suggested that estrogens stimulate the process of gamete release and coral spawning, and that their eggs contain estrogenic compounds that will aid in their final maturation^27,31^. It is also speculated that differences in estrogen concentration will regulate the reproductive processes of corals^32^. Tarrant et al.^33^ found that corals have the capacity to take up estrogens from the water and accumulate these compounds in their tissue, although the consequences of this bioaccumulation are unknown.

Corals are metaorganisms, i.e., a biological unit that includes the host and its associated microbiome, whether stable or transitory^34^. Coral-associated microbes are diverse, complex, dynamic, and essential for the functioning and balance of the coral reef ecosystem^35–38^. These microorganisms have several functions that aid in coral fitness^36,37,39,40^, such as nitrogen fixation^41^, ammonia metabolism^42^, removal of nitrogenous waste^42^, production of antibiotics, physical occupation of coral space, and competition with other microorganisms, in order to protect the host from invasion of opportunistic pathogens^37,43,44^.

The coral-associated microbial community is sensitive to environmental changes and can quickly respond and adapt to new environmental conditions, which is important in maintaining the homeostasis of the metaorganism^37,42,44^. Different environmental stressors, such as thermal stress^38,45,46^, oil^47,48^ the presence of pathogens^40,49,50^ or ocean acidification^51,52^ can cause shifts in the coral microbiome, which may change their diversity, abundance and functionality for long periods of time^53^. For example, exposure of the coral *Acropora muricata* to relatively high levels of nickel and copper resulted in coral bleaching. Copper pollution also modified the coral microbiome (eukaryotes and prokaryotes)^54^. One study showed that the bacterial core associated with corals can respond to a gradient of anthropogenic pollution^55^. Different stressors, such as climate change, pollution and overfishing are correlated with an increase in the richness and diversity of specific coral-associated microbes, including the commonly found association of higher abundances of members of Vibrionaceae and Altermonadaceae with stressed and diseased corals^56–58^. Further, coral micro-organisms are able to degrade or neutralize toxic substances that threaten coral health, suggesting that the microbiome can protect coral health and mitigate the effects of pollution^47,59^. However, no reports about the effects of EDCs on the health of corals and their microbiome are currently available.

Here, we evaluated the effects of EE2 on the coral *Mussismilia harttii* in a microcosm system, and, for the first time, the impacts of an EDC on a coral-associated microbiome.

## Results

### Visual health status and ***F***_v_/***F***_m_

**Figure 1:**
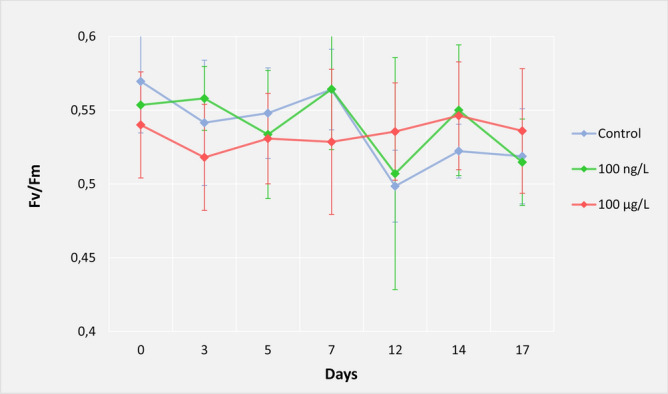
Measurements of *F*_v_/*F*_m_ in *Mussismilia harttii* during the 17 days of experiment, with the treatments: control (without EE2), 100 ng L^−1^ and 100 µg L^−1^ of EE2 (n = 4).

No significant variations in water-quality parameters (temperature, salinity and pH) (Supplementary Table S1) or visual changes (Fig. S1) were observed during the experiment. These observations agreed with lack of change in the *F*_*v*_*/F*_*m*_ ratios. At the beginning of the experiment (sampling time T0), *F*_*v*_*/F*_*m*_ = 0.569 in the control samples, 0.553 in the 100 ng L^−1^ and 0.540 in the 100 µg L^−1^ samples. The ratio of *F*_*v*_*/F*_*m*_ in all samples did not change over time, remaining at about 0.537, above the healthy *F*_*v*_*/F*_*m*_ value (> 0.5) (Fig. 1). After normalization of the *F*_*v*_*/F*_*m*_ data, no significant difference was apparent among the samples (either in the sampling times or in treatments with or without EE2).

### Coral microbiome data analyses

To investigate the microbial profile of corals exposed to two different EE2 concentrations and in a control treatment (i.e., without EE2), the 16S rRNA gene was sequenced and analyzed. A total of 2,603,433 sequences, ranging from 162,262 to 11,409 sequences per sample, were obtained. A large number of sequences were assigned to mitochondrial DNA from the host coral and were removed from the dataset. Quality trimming and data normalization yielded 104,640 sequences, corresponding to 2180 sequences per sample. This sequencing depth was enough to evaluate the total microbiome patterns, as shown in the rarefaction curve (Fig. S2), establishing that most of the microbiome was covered by the sequencing. The diversity and richness of the bacterial community were calculated based on the numbers of OTUs, using the Chao1 and Shannon indexes. The numbers of OTUs of the samples varied over the experiment; at the end, the samples from the 100 µg L^−1^ EE2 treatment had the most OTUs, followed by the control and 100 ng L^−1^ EE2 (Fig. S3A). A two-way ANOVA of the OTUs indicated a significant influence of EE2 (p = 0.02), with the number of OTUs in the 100 µg L^−1^ EE2 treatment higher than in the control. The 100-ng treatment did not differ from the other treatments, nor did time have a significant influence. The Shannon index showed no significant difference between any factors (Fig. S3B).

**Figure 2:**
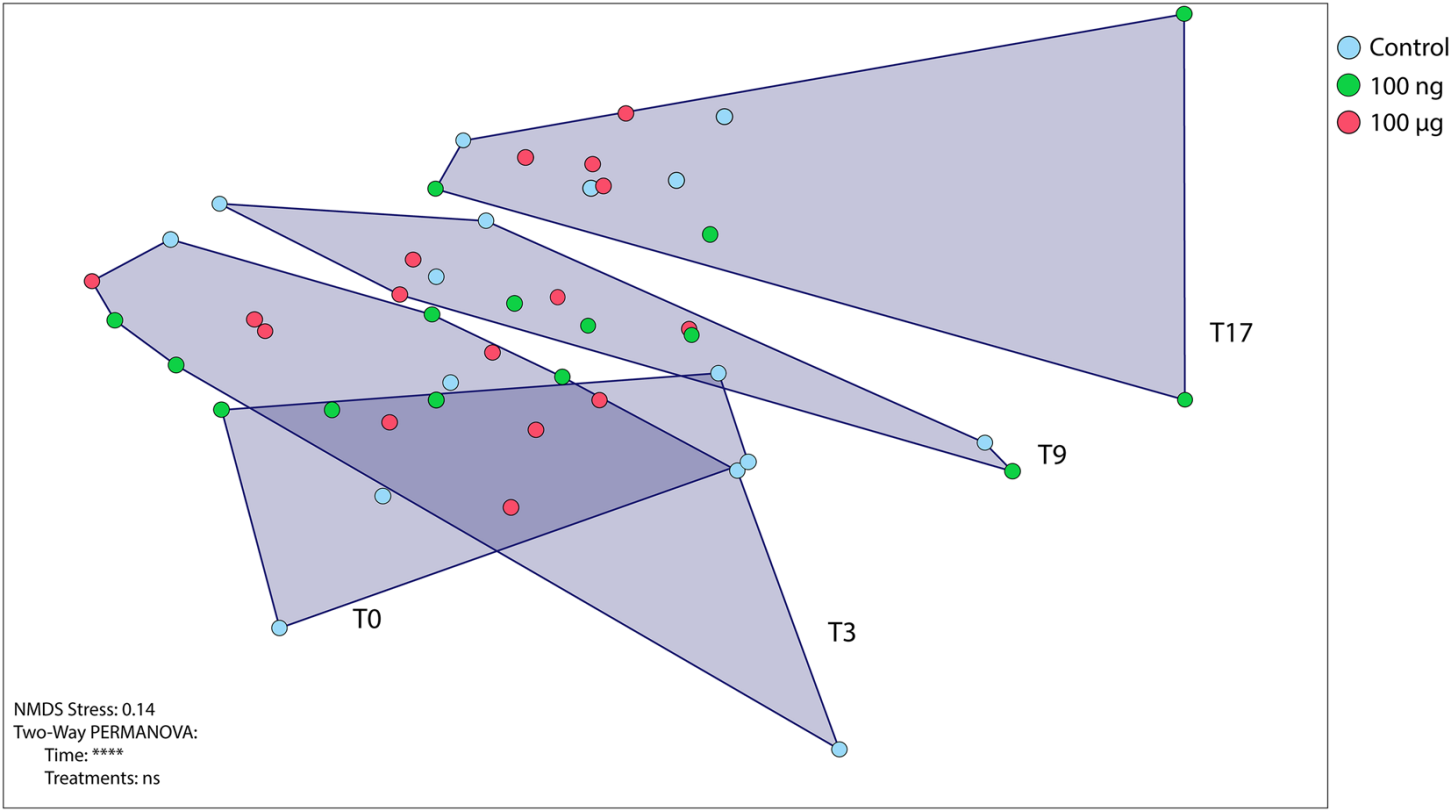
Non-metric multidimensional scaling (NMDS) ordination using Bray–Curtis dissimilarity, based on abundances of OTUs in samples from *Mussismilia harttii* treatments (n = 4).

**Figure 3:**
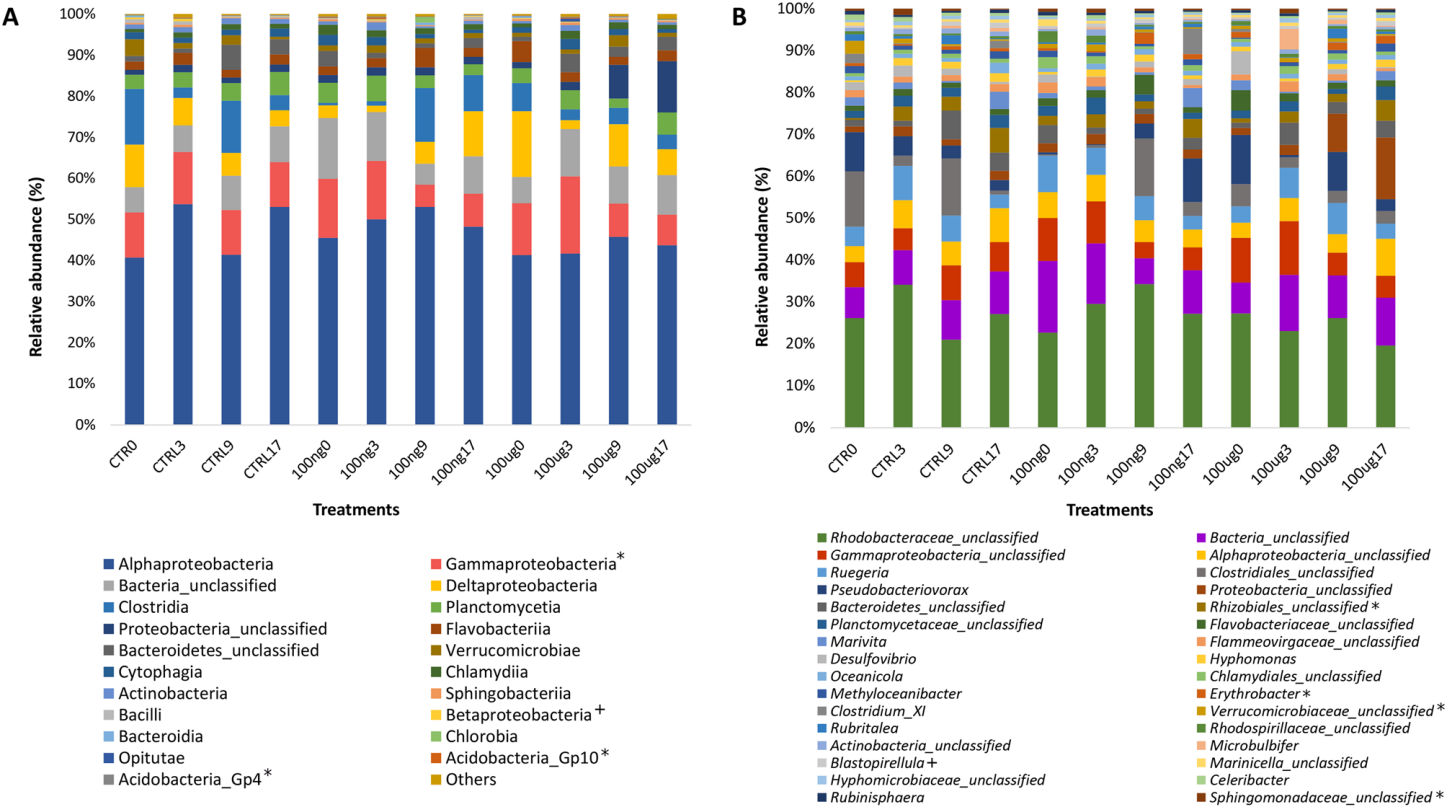
(**A**) Taxonomic classification and relative abundance of the bacterial classes from *Mussismilia harttii* treatments: control (without EE2), 100 ng L^−1^ and 100 µg L^−1^ of EE2 (n = 4). + indicates significant difference between times (days; T3 and T9) and treatment control with 100 µg L^−1^ of EE2; *indicates significant difference between times (days; T3, T9 and T17 in Gammaproteobacteria) and T3 and T17 in Acidobacteria. (**B**) Taxonomic classification and relative abundance of bacterial genera from *M. harttii* treatments: control (without EE2), 100 ng L^−1^ and 100 µg L^−1^ of EE2 (n = 4). + indicates significant difference between times (days; T0 and T17) and interaction among factors; *indicates significant difference between times. The third day (T3) was significantly different in Sphingomonadaceae_unclassified; times T0 and T17 in Verrucomicrobiaceae_unclassified; times T3 and T9 in *Erythrobacter*; and in Rhizobiales_unclassified, times T0 and T17 differed significantly, as did T9 and T17.

NMDS ordination of the OTU results demonstrated that, despite a slight trend toward treatment-based clustering at the last sampling time, the total coral-associated microbiome was not significantly affected by EE2 contamination, and was clustered only by time (days 0, 3, 9 and 17, i.e. T0, T3, T9 and T17 respectively) (p < 0.0001, pseudo F = 1.7321), regardless of the treatment (Fig. 2). Taxonomic investigation showed that Proteobacteria was the most abundant phylum in the coral samples, with or without estrogen. This group comprised about 60% of the bacterial community in all samples, followed by unclassified Bacteria, Firmicutes and Bacteroidetes. Additionally, Acidobacteria showed significant differences between times T0 and T17, as did Deinococcus-Thermus, which also showed interaction among factors, according to ANOVA followed by a Tukey test (p < 0.05) (Fig. S4). At the class level, Alphaproteobacteria predominated, comprising about 40% of the total microbiome. Betaproteobacteria showed a significant reduction in relative abundance compared to the control with 100 μg of EE2 (Two-Way ANOVA, p < 0.05) and Gammaproteobacteria and Acidobacteria groups GP4 and GP10 showed significant differences as a function of the incubation time (Two-Way ANOVA, p < 0.05) (Fig. 3A). At the genus level, taxonomic diversity and relative abundance were similar (with few differences) among all samples. Rhizobiales_unclassified, Verrucomicrobiaceae_unclassified, Sphingomonadaceae_unclassified, *Erythrobacter*, and *Blastopirellula* differed significantly among the different collection times; the last genus showed interaction among the factors (Fig. 3B). Most genera were unclassified, related to Rhodobacteriaceae, Bacteria, Gammaproteobacteria, Alphaproteobacteria, *Ruegeria*, Clostridiales and *Pseudobacteriovorax*.

**Figure 4:**
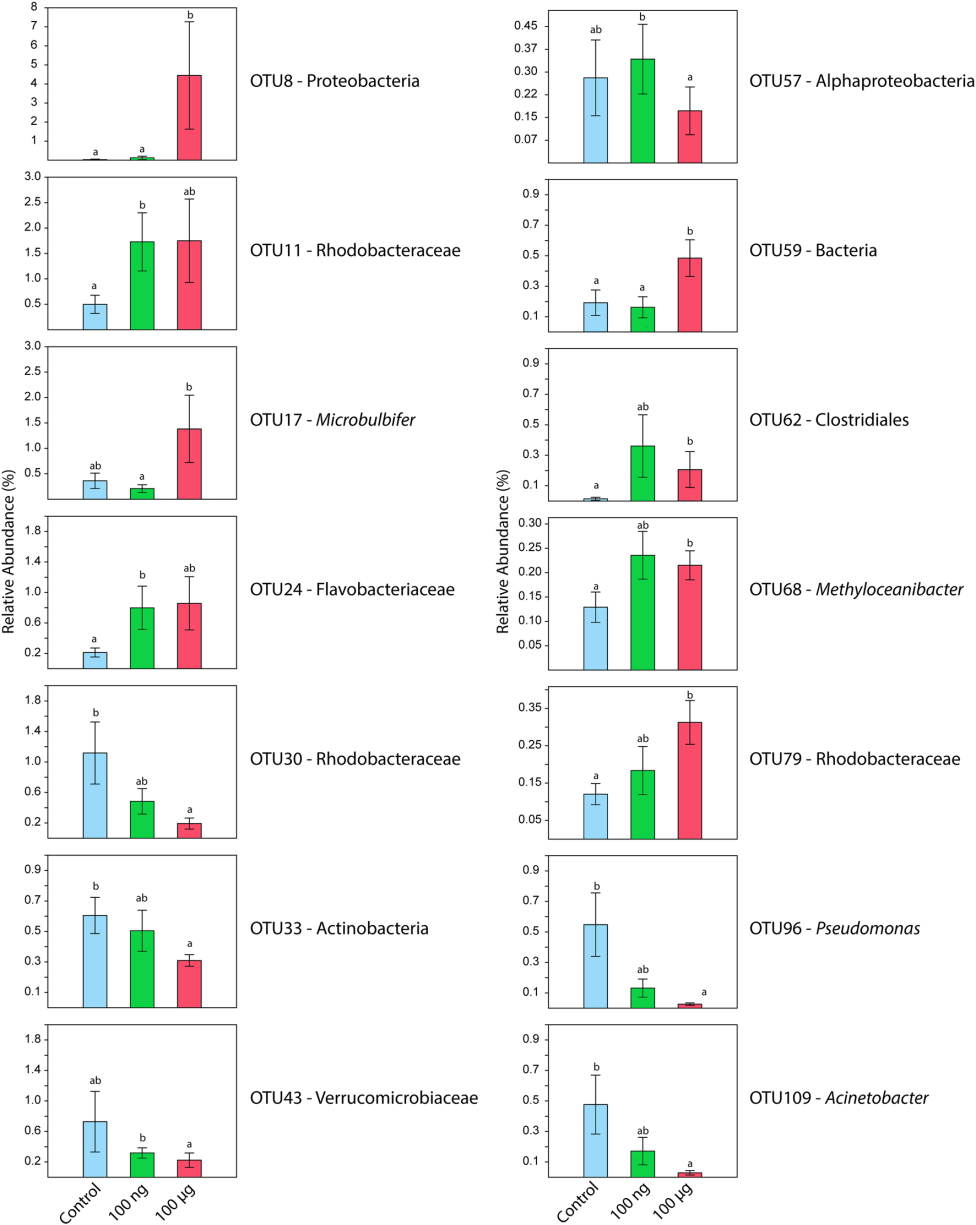
Indicator Species Analysis for *Mussismilia harttii* treatments, showing the relative abundance of the bacterial groups in each treatment (control, 100 ng L^−1^ and 100 µg L^−1^ of EE2).

An Indicator Species Analysis (ISA) was performed with the 115 most abundant OTUs (all OTUs with more than 0.1% of the global relative abundance), to assess the impacts of EE2 contamination on specific organisms. We found 14 OTUs that were significant for at least one treatment. OTU8-Proteobacteria, OTU59-Bacteria, OTU62-Clostridiales, OTU68-*Methyloceanibacter*, and OTU79-Rhodobacteraceae were more abundant in the EE2 treatments than in the control. OTU30-Rhodobacteraceae, OTU33-Actinobacteria, OTU96-*Pseudomonas* and OTU109-*Acinetobacter* were more abundant in the control than in 100 µg L^−1^ EE2 (Fig. 4).

## Discussion

Marine pollution and environmental stressors threaten coral reefs and wildlife worldwide^60,61^. Coral reefs in coastal areas are widely exposed to contaminated seawater from untreated effluents, exploratory marine activities, metals, human actions, climate change and spills of oil and other substances^62–65^. Corals and other cnidarians can be exposed to synthetic compounds such as estrogens by absorbing dissolved products, ingesting food, or contacting sediments or suspended solids^66^. They are especially sensitive to lipophilic compounds because their lipid-rich tissues facilitate absorption^67^. Ethinylestradiol, a synthetic estrogen of the endocrine-disruptor group, is persistent in the marine ecosystem, is difficult to degrade, and can accumulate in animal tissues^68^. For this reason, it is important to determine how EE2 pollution could affect coral reefs. This study pioneered in investigating the specific effects of estrogenic pollution on the health of corals and their associated microbiome.

Our experiment was carried out in a microcosm system, which proved to be efficient for testing the selected conditions. Physical–chemical measurements (temperature, pH and salinity) did not vary significantly and were not affected by the estrogen contamination, even at the highest concentration (100 µg L^−1^). Our results showed that over the 17 days of the experiment, the coral health status did not change visually, with no bleaching or tissue loss compared to the control and to the status at the beginning of the experiment. This agreed with the *F*_*v*_*/F*_*m*_ measurements, which showed a mean of 0.537 in all treatments (including the control), which is above the level for healthy conditions (> 0.5)^69^ and is similar to the level found by Santos and colleagues (2014)^46^ in control samples. An *F*_v_/*F*_m_ ratio of around 0.600 was observed in *M. harttii* at 25 °C; when this coral was exposed to a higher temperature (27.3 °C) and contamination by 3.8 µg L^−1^ of copper, the ratio decreased significantly, negatively affecting the coral^70^. The impacts of EE2 on coral-associated algae have not yet been investigated. Our results for *F*_*v*_*/F*_*m*_ showed that in the concentrations used (100 ng and 100 µg L^−1^), EE2 did not affect the algal photosynthetic efficiency, suggesting a lack of a major impact on the metaorganism health. The use of pulse-amplitude-modulated (PAM) fluorometry is an indirect method used as a proxy for coral health, based on the photosynthetic efficiency of Symbiodiniaceae^40,47,71–73^.

To investigate whether EE2 pollution could affect the microbiome, the microbial profile of the coral samples was analyzed. The diversity and richness indexes varied among samples and sampling times (T0, T3, T9 and T17). The richness of OTUs from the 100 µg L^−1^ EE2 treatment differed significantly from the other samples (control and supplemented with 100 ng L^−1^ EE2), suggesting that higher concentrations of estrogens, on the order of µg L^−1^, can affect the coral-associated microbiome. The number of OTUs in all samples fluctuated during the experiment. The number of OTUs from the control samples increased until a slight decrease on the last day. The OTU numbers increased in samples with 100 ng L^−1^ EE2 in the first 3 days and then decreased, finishing the experiment with the lowest number; whereas samples with 100 µg L^−1^ EE2 showed the opposite behavior, with a decrease in the first 3 days and then an increase in OTU numbers until the end of the experiment, when the number of OTUs reached its highest level (Fig. S3a). A possible reason for this increase in the number of OTUs is that certain bacteria can use EE2 as a carbon source, and therefore the presence of EE2 can create new ecological niches, supporting a richer microbial community.

Microbial indicators of pollutants have been described in aquatic ecosystems, such as mangroves^76,77^ and seawater^78,79^. Because of their plasticity and rapid responses, microorganisms are early indicators of environmental changes and may be indicators of environmental quality^80,81^. The coral-microbiome association could be a key factor in the functioning and organization of marine ecosystems^82^. In some cases, these microbes contribute to the metaorganisms’ resilience to impacts though their rapid response to environmental disturbances, helping to mitigate stress^45^. In the present study, EE2 contamination (100 ng or 100 µg L^−1^) did not cause significant changes in the total microbial diversity, as shown in the NMDS analysis. A slight trend toward correlation between replicates of the same treatments was observed by day 17. The dominant major microbial groups observed in our samples are similar to those previously reported as associated with *Mussismilia harttii*^46,47,74,75^.

Although the taxonomic results did not indicate that EE2 impacted the total coral microbiome, the Indicator Species Analysis (ISA) showed that certain bacterial groups were enriched in the presence or absence of EE2 contamination. OTU8 (Proteobacteria) was enriched about sevenfold in 100 µg L^−1^ EE2-contaminated samples but nearly absent in other conditions. OTU17-*Microbulbifer* and OTU59-Bacteria behaved similarly, with fourfold higher abundances in 100 µg L^−1^ EE2-contaminated samples than in the control and 100 ng L^−1^ EE2. OTU79-Rhodobacteraceae, a family that includes estrogen degraders^83–86^, was most abundant in samples contaminated with 100 µg L^−1^ EE2. OTU68-*Methyloceanibacter* was also significantly more abundant in EE2-contaminated samples, which may be related to these microorganisms’ ability to degrade steroids and also to produce the enzyme cholesterol oxidase^87^. A recent study found a strong correlation between estrogen metabolism and members of Clostridia^88^, which could explain the statistical predominance of OTU62-Clostridiales in 100 µg L^−1^ EE2-exposed corals. The high amount of estrogen in this treatment could be selecting microbial groups that can use estrogens as an energy/carbon source. Likewise, OTU96-*Pseudomonas*, OTU109-*Acinetobacter*, OTU30-Rhodobacteraceae and OTU43-Verrucomicrobiaceae were significantly sensitive to EE2 contamination at concentrations of 100 ng L^−1^ and 100 µg L^−1^, and were positively correlated with control samples (without EE2). These groups can be considered indicators of an absence of EE2 contamination at these concentrations, and should be further investigated as potential Beneficial Microorganisms for Corals (BMCs)^37^ to mitigate the harmful effects of EE2.

Although the physiological effects of coral exposure to estrogen are still underexplored, estrogens may be active in invertebrate gametogenesis, increasing their concentration in seawater and in coral eggs during spawning^27^. Corals are able to take up estrogens from water at low concentrations, on the order of pg L^−1^, and the true effects and mechanisms of action of these micropollutants are unknown^8,28,30^. It is suggested that steroidal estrogens may influence and aid in coral reproduction^26^, and these estrogens have been designated as important bioregulators of the metabolism of scleractinian corals^31^. Although no harmful effects were observed in our experiment, the effects of endocrine disruptors may appear after long-term exposure to low concentrations^5^, and therefore longer-term experiments may be necessary to elucidate their effects. It was possible, however, to identify specific coral-associated microbial indicators of EE2 contamination. Also, it is important to consider other responses from corals, to elucidate the impact of EE2 on their health, such as reproductive ability, lipid and protein contents, calcification and respiration rate. These results suggest that specific levels of EE2 contamination might affect the microbiome of the coral metaorganism within short periods of time, and that these groups are early indicators of this contamination.

## Material and methods

### Coral sample

*Mussismilia harttii* corals were collected from Santa Cruz Cabrália, Coroa Vermelha Reefs, Brazil in January 2017, under permit number 56537-1 from the Brazilian Environmental Agency (ICMBio/SISBIO). Pieces of this endemic Brazilian coral were collected haphazardly, in triplicate, with a hammer and chisel by SCUBA diving at 8 m depth at three stations (16° 20′ 57.99′′ S; 038° 58′ 45.00′′ W; 16° 20′ 39.30′′ S; 038° 58′ 38.10′′ W; 16° 22′ 2.20′′ S; 039° 00′ 15.63′′ W), and were immediately stored in individual clean plastic bags until arrival in Rio de Janeiro for microbiome analysis. The microcosm experiment was performed in the Marine Aquarium of Rio de Janeiro (AquaRio) in March 2017, and the data were analyzed in the Molecular Microbial Ecology Laboratory (Federal University of Rio de Janeiro).

### Microcosm experimental design

**Figure 5:**
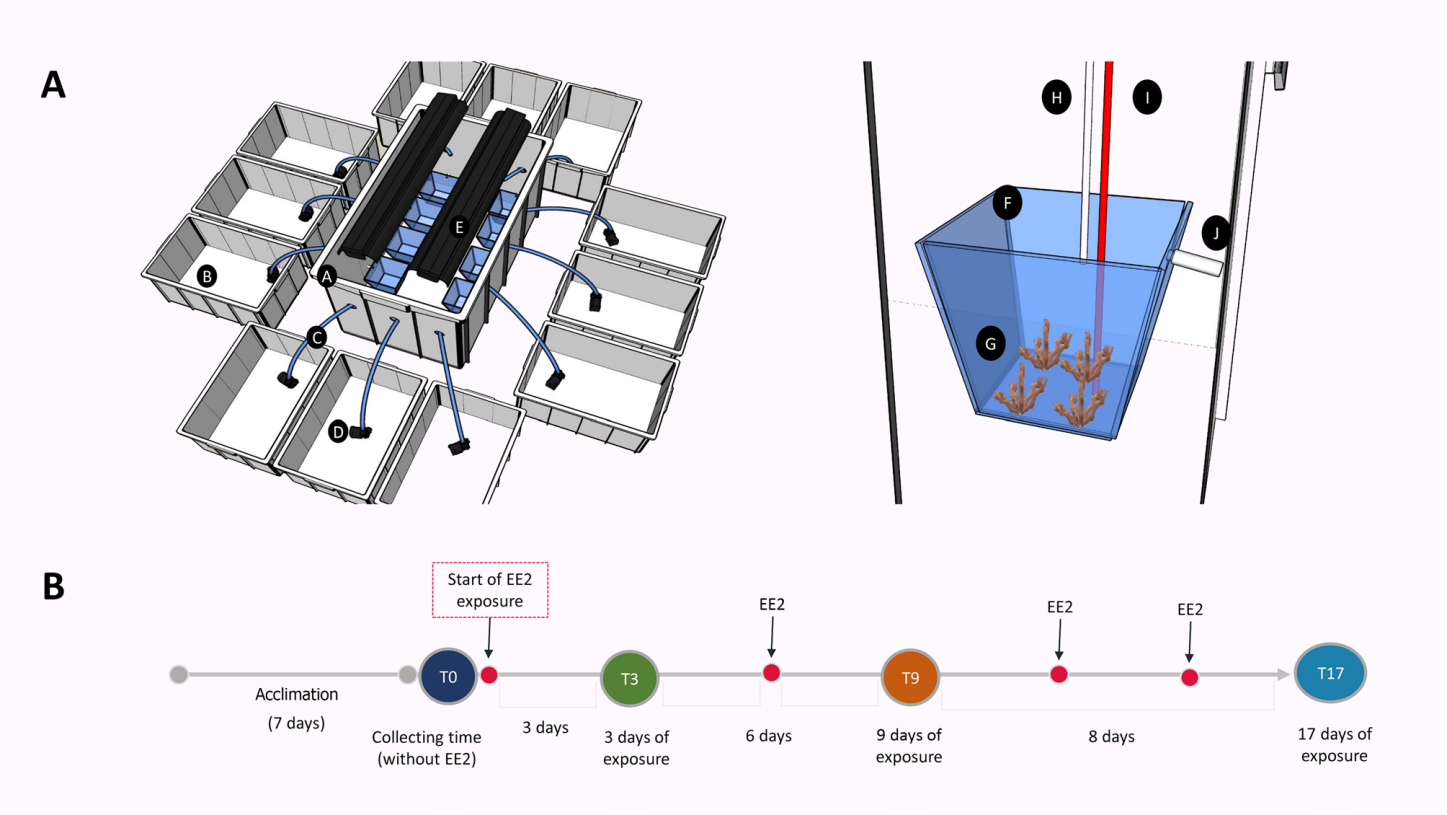
(**A**) Schematic representation in 3D modeling of the microcosm, using the software SketchUp. The letters indicate the parts of the structure. A. Water-bath; B. Treatment sumps; C. Aquarium supply hose; D. Circulation pump; E. Illumination system (2 fluorescent lamps and 2 blue lamps); F. Aquarium; G. Four fragments of *Mussismilia harttii*; H. Aquarium supply hose; I. Air entrance hose; J. Overflow. (**B**) Timeline showing an overview of the experiment.

The microcosm was constructed of 12 1.2-L aquariums, with individual sumps (10 L) used for seawater circulation and oxygenation, in a recirculating system. Each sump had a submersible pump (Mini A, Sarlo Better, São Caetano do Sul, Brazil) connected to a hose to recirculate the water between the aquarium and its dedicated sump at a turnover of 170 L h^−1^ (Fig. 5). Each treatment consisted of four replicate aquariums, each containing four randomly distributed *M. harttii* polyp branches (~ 6 cm). *M. harttii* has large separate trumpet-like polyps, which facilitates treating them individually. An air-bubbling system was used to provide a turbulent flow, suitable for the corals, in each replicate. After 7 days of acclimatization, the treatments were started, consisting of 4 replicates supplemented with 100 ng L^−1^ of EE2, 4 with 100 μg L^−1^ of EE2, and 4 control aquariums (without hormone). The replicate aquarium treatments were randomly distributed in a controlled-temperature water bath, operated at 25 °C for the 17-day experiment. The water-bath temperature was controlled with Full Gauge MT-518ri thermostats (Canoas, Brazil). The aquariums were filled with seawater collected 10 km offshore from Rio de Janeiro, and 30% of the water was changed with unfiltered seawater from sumps every 2 days, with complementary addition of EE2 to maintain the experimental concentration when applicable. The microcosm was artificially illuminated with two actinic blue tubes (T5 24″, 24 W) and six fluorescent Power Compact bulbs (FLC, 20 W) in a 12-h photoperiod regime, resulting in an irradiance of 400 μmol photons m^–2^ s^−1^. The corals were not fed during the experiment. Figure 5 shows a schematic representation of the microcosm.

### Physical–chemical parameters

Temperature, pH and salinity were measured every 2 days in each aquarium, using a YSI 550A probe.

### Evaluation of coral health parameters

Coral holobiont health was assessed based on the maximum quantum yield of the zooxanthellae-associated Photosystem II (*F*_*v*_*/F*_*m*_)^89^ and the apparent health status during the experiment, including by photographs. The color of the coral tissue was observed visually, by comparing photographs taken at T0, T3, T9 and T17. The Coral Health Chart (University of Queensland) was used to compare the photographs of corals in the different treatments with or without EE2 during the experiment^90^.

The photosynthetic parameters were evaluated throughout the experiment, using a Diving-PAM pulse amplitude modulated underwater fluorimeter (Heinz Walz GmbH, Germany), with the following configuration: Measuring Light Intensity (MI) = 5; Saturation Pulse Intensity (SI) = 8; Saturation Pulse Width (SW) = 0.8; Gain (G) = 1; Damping (D) = 1. The measurement was performed using a Fiber Quantum Sensor (diameter 1 mm) connected to the Diving-PAM after 20 min of dark acclimatization. The results were analyzed by two-way ANOVA, using Past 3.25 software^91^.

### Coral microbiome investigation

Polyps of *Mussismilia harttii* were collected from each aquarium at times (days) T0, T3, T9 and T17 for analysis of the bacterial community and the EE2 impact on the corals microbiome. The coral polyps were fragmented and stored in cryotubes, flash-frozen in liquid nitrogen. Subsequently, the samples were macerated using a mortar and pestle, and the total DNA was extracted from 0.5 g of sample, using a Qiagen DNeasy Power Soil kit (Qiagen, Hilden, Germany), according to the manufacturer’s instructions. The DNA was quantified with a Qubit 2.0 Fluorometer High-Sensitivity DNA Kit (Invitrogen, USA) and stored at − 80 °C.

The V4 variable region of the 16S rRNA from the *M. harttii* samples was amplified using 515F/806R primers^92^. About 10 ng of DNA was used for PCR amplification and paired-end sequencing by a single-step 30-cycle PCR, using a HotStarTaq Plus Master Mix Kit (Qiagen, USA). The PCR conditions were: 94 °C for 3 min, followed by 28 cycles at 94 °C for 30 s, 53 °C for 40 s and 72 °C for 1 min, with a final elongation step at 72 °C for 5 min. The samples were sequenced at the Argonne National Laboratory (http://ngs.igsb.anl.gov, Lemont, IL, USA) through the Next Generation Sequencing Core on an Illumina Miseq (Illumina, San Diego, CA, USA), following the manufacturer’s guidelines.

The raw sequences were processed using Mothur v.1.39.1 software^93^ in order to assess the total bacterial diversity of the samples. Paired-end sequences were joined using the make.contigs command with checkorient = t. The sequences were then screened with the screen.seqs command, removing those outside the size range of 245–300 nucleotides and/or with any ambiguity and/or with homopolymers longer than 8. Sequences were then aligned against a pre-processed version of the Silva NR database (passed through a virtual PCR with the same primers used to amplify the samples). The resulting alignment was submitted to screen.seqs and filter.seqs to remove sequences with poor alignment and uninformative columns of the alignment. Then, sequences were pre-clustered using the pre.cluster command with parameter diffs = 2. Chimeric sequences were detected using the chimera.uchime command, using the sequences themselves as a reference, with the option derreplicate = t. Sequences were classified using the Greengenes database (version from August 2013), employing an 80% confidence threshold, and those classified as chloroplasts, mitochondria, Archaea, Eukarya, or not assigned to any kingdom were removed. The remaining high-quality sequences were clustered into operational taxonomic units (OTUs) using dist.seqs followed by the cluster command, with a 3% sequence dissimilarity cutoff, and all singletons were removed. Last, the samples were randomly normalized to the same number of sequences (2180). The OTU distribution in each sample was used to determine the bacterial community diversity and richness, as well as to analyze the microbial structure, using Non-metric multidimensional scaling (NMDS) ordination with Bray–Curtis distance. A two-way permutational multivariate analysis of variance (two-way PERMANOVA) was performed to test for the impact of time and EE2 contamination on OTU distribution. All statistical tests were done using PAST 3.25 software^91^. A blocked Indicator Species Analysis (ISA) was performed to determine microbial indicator groups. The OTUs that were significantly impacted (p < 0.05) and had an indicator value > 60 are shown. This analysis was conducted with PC-ORD 6.0 software^94^. The data generated were deposited in the NCBI Sequence Read Archive (SRA) and are available under accession number PRJNA543294.

## Supplementary Information

Supplementary Information 1.

Supplementary Information 2.
