# Supplementary material for: Estrogen induces shift in abundances of specific groups of the coral microbiome
Source: Sci Rep. 2021 Feb 2;11:2767. doi: 10.1038/s41598-021-82387-x (PMC7854615; doi:10.1038/s41598-021-82387-x)
Supplement: Supplementary file 1 — Supplementary Information 1. [file 41598_2021_82387_MOESM1_ESM.pdf]

Supplementary Material

Estrogen induces shift in abundances of specific groups of the coral microbiome

Caren L.S. Vilela, Helena D.M. Villela, Gustavo A.S. Duarte, Erika P. Santoro, Caio T.C.C. Rachid, Raquel S. Peixoto

Figure S1. Organization of the aquariums and treatments: control, 100 ng L^{-1} and 100 µg L^{-1} of EE2, with the coral's visual health status after 0, 3, 9 and 17 days (T0, T1, T2 and T3).

Figure S2. Rarefaction curves of 16S rRNA sequences obtained by Illumina MiSeq sequencing from coral samples (control and treatments with EE2)**.** Colored lines represent the OTUs in the samples.

Figure S3. A. Numbers of OTUs from coral samples during 17 days of experiment. **B.** Shannon index value during 17 days of experiment on the coral samples.

Figure S4. Taxonomic classification and relative abundance of the bacterial phyla from *Mussismilia harttii* treatments: control (without EE2), 100 ng L^{-1} and 100 µg L^{-1} of EE2 (n = 4). *: significative difference between time 0 and 17; ¥: significative difference between time 0 and 17 and interaction among factors.

Table S1. Physical-chemical parameters of the aquarium water at each sampling time, measured with a YSI 550A probe.

Table S2: Results of the PERMANOVA analysis based on the Bray-Curtis similarity matrix for the microbiome of corals treated or not with EE2.

Figure S1

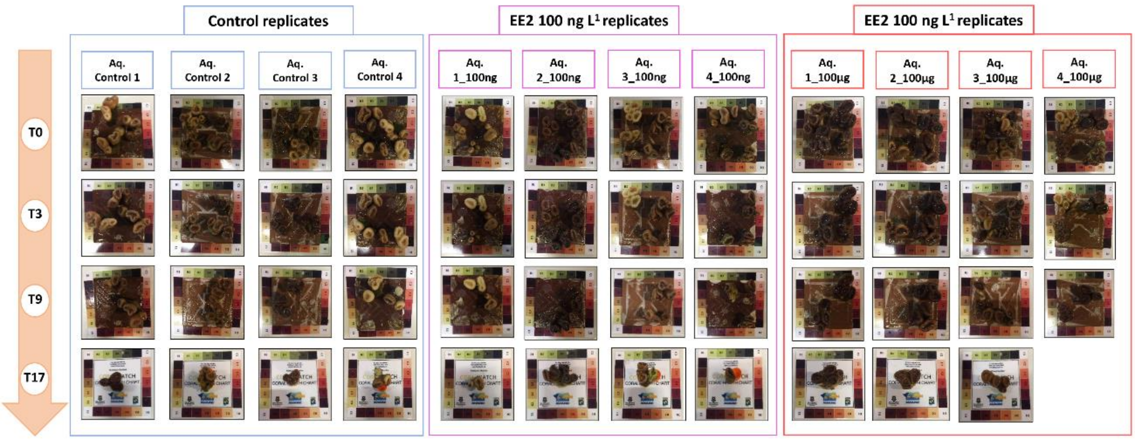

Control replicates
EE2 100 ng L1 replicates
EE2 100 ng L1 replicates
Aq. Control 1
Aq. Control 2
Aq. Control 3
Aq. Control 4
Aq. 1_100ng
Aq. 2_100ng
Aq. 3_100ng
Aq. 4_100ng
Aq. 1_100µg
Aq. 2_100µg
Aq. 3_100µg
Aq. 4_100µg
T0
T3
T9
T17

Figure S2

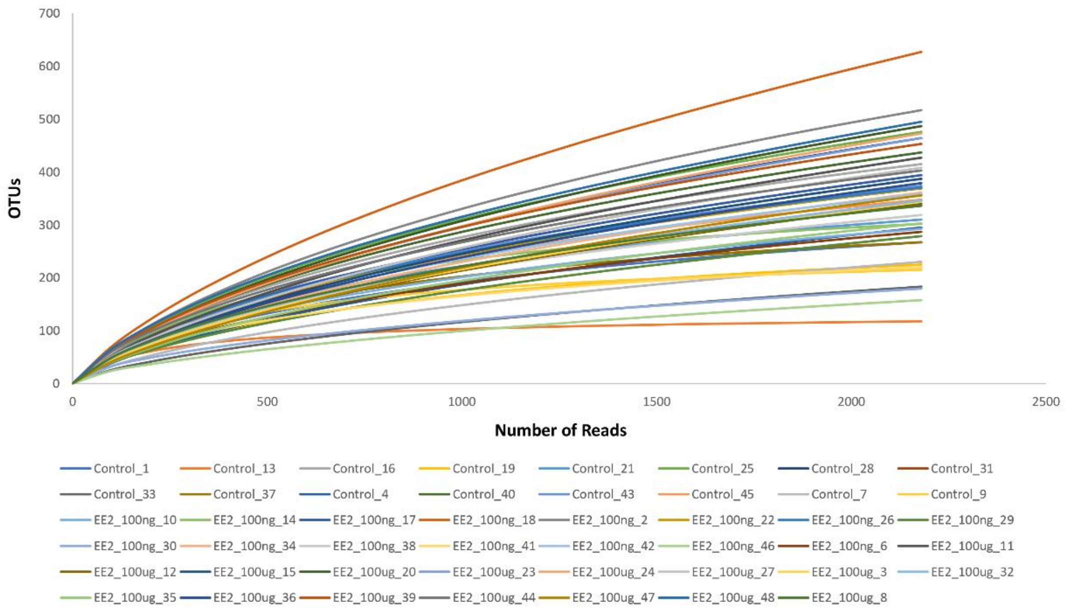
OTUs
Number of Reads
700
600
500
400
300
200
100
0
0
500
1000
1500
2000
2500
Control_1
Control_13
Control_16
Control_19
Control_21
Control_25
Control_28
Control_31
Control_33
Control_37
Control_4
Control_40
Control_43
Control_45
Control_7
Control_9
EE2_100ng_10
EE2_100ng_14
EE2_100ng_17
EE2_100ng_18
EE2_100ng_2
EE2_100ng_22
EE2_100ng_26
EE2_100ng_29
EE2_100ng_30
EE2_100ng_34
EE2_100ng_38
EE2_100ng_41
EE2_100ng_42
EE2_100ng_46
EE2_100ng_6
EE2_100ug_11
EE2_100ug_12
EE2_100ug_15
EE2_100ug_20
EE2_100ug_23
EE2_100ug_24
EE2_100ug_27
EE2_100ug_3
EE2_100ug_32
EE2_100ug_35
EE2_100ug_36
EE2_100ug_39
EE2_100ug_44
EE2_100ug_47
EE2_100ug_48
EE2_100ug_8

Figure S3

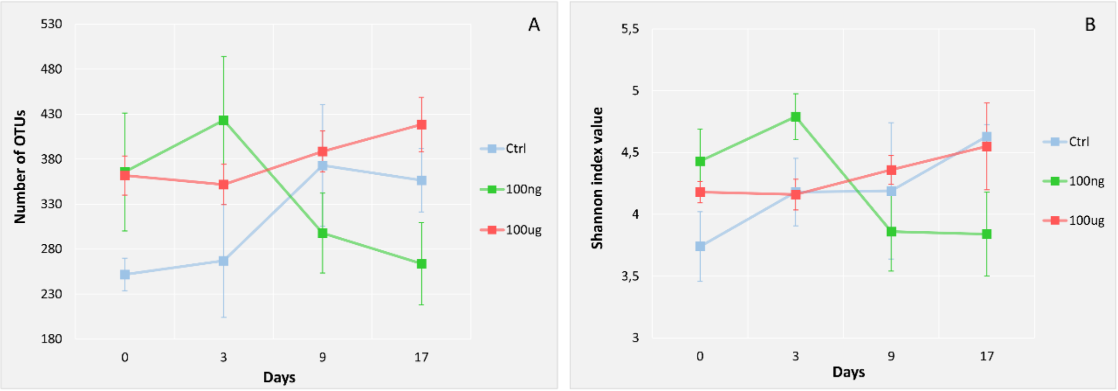

A
Number of OTUs
530
480
430
380
330
280
230
180
0
3
9
17
Days
Ctrl
100ng
100ug
B
Shannon index value
5,5
5
4,5
4
3,5
3
Days

Figure S4

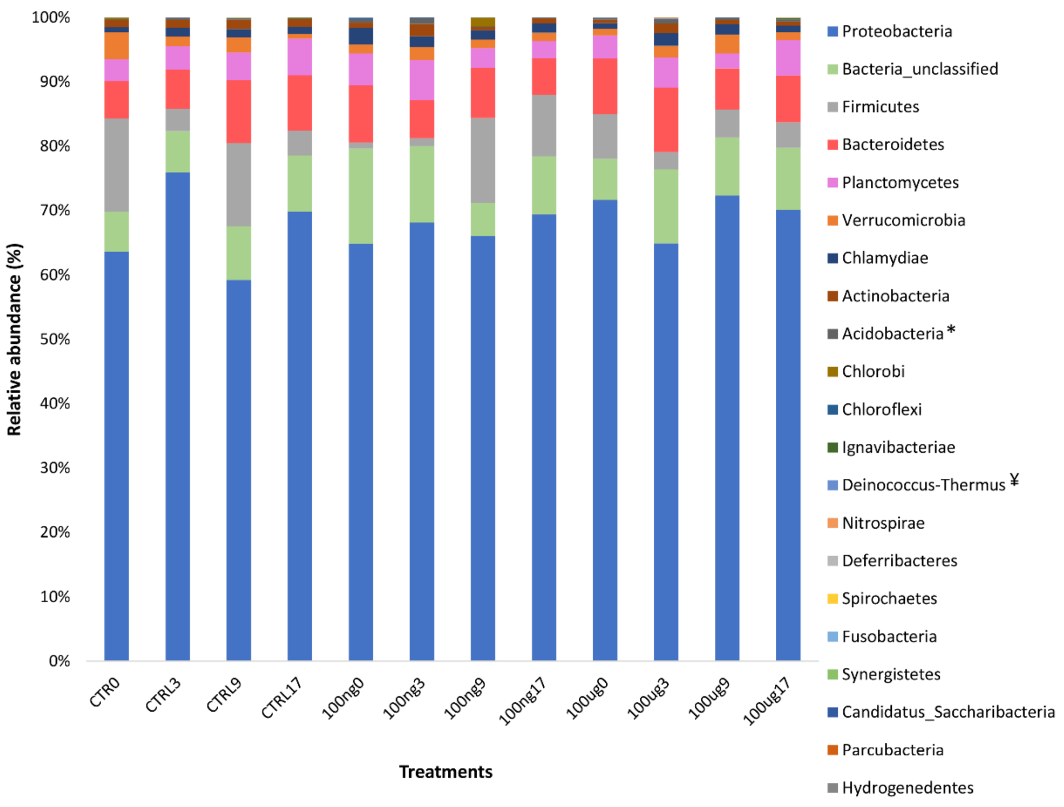

For a better view of the data, follow the link for accessing the OTU abundance table: D:\Arquivos novos CORAL-EE2\OTU table abundance.xlsx

Table S1

Sampling time	Treatment (Replicate)	Temperature	pH	Salinity
T0	Control (1)	25.5	8.1	35
	EE2 100 ng (2)	25.3	8.1	35
	EE2 100 µg (1)	25.4	8.1	35
	Control (2)	25.7	8.1	35
	EE2 100 ng (1)	25.3	8.1	35
	EE2 100 ng (4)	25.1	8.1	36
	Control (3)	25.5	8.1	35
	EE2 100 µg (3)	25.3	8.1	36
	Control (4)	25.5	8.0	37
	EE2 100 ng (3)	25.1	7.9	36
	EE2 100 µg (2)	25.3	8.1	34
	EE2 100 µg (4)	25.4	8.1	36
T3	Control (1)	25.4	8.0	35
	EE2 100 ng (2)	25.3	8.2	35
	EE2 100 µg (1)	25.5	8.1	35
	Control (2)	25.5	8.1	34
	EE2 100 ng (1)	25.6	8.1	35
	EE2 100 ng (4)	25.4	8.1	35
	Control (3)	25.5	8.1	34
	EE2 100 µg (3)	25.3	8.1	35
	Control (4)	25.5	8.1	34
	EE2 100 ng (3)	25.3	8.1	35
	EE2 100 µg (2)	25.3	8.1	35
	EE2 100 µg (4)	25.5	8.3	35
T9	Control (1)	25.3	8.1	36
	EE2 100 ng (2)	25.4	8.1	36
	EE2 100 µg (1)	25.4	8.2	35
	Control (2)	25.4	8.1	35
	EE2 100 ng (1)	25.6	8.2	37
	EE2 100 ng (4)	25.6	8.2	37
	Control (3)	25.4	8.1	37
	EE2 100 µg (3)	25.3	8.2	36
	Control (4)	25.2	8.2	37
	EE2 100 ng (3)	25.2	8.1	36
	EE2 100 µg (2)	25.5	8.2	36
	EE2 100 µg (4)	25.3	8.0	35
T17	Control (1)	26.9	8.2	34
	EE2 100 ng (2)	27.0	8.3	35
	EE2 100 µg (1)	26.9	8.2	35
	Control (2)	27.0	8.2	35
	EE2 100 ng (1)	26.9	8.2	35
	EE2 100 ng (4)	26.9	8.3	35
	Control (3)	26.8	8.2	35
	EE2 100 µg (3)	26.8	8.2	34
	Control (4)	26.7	8.2	35
	EE2 100 ng (3)	27.1	8.2	34
	EE2 100 µg (2)	26.9	8.2	35
	EE2 100 µg (4)	26.9	8.2	35

Table S2

Source	Sum of sqrs	df	Mean square	F	p
**Time**	1.389	3	0.46302	1.7321	0.0001*
**Tratamento**	0.44002	2	0.22001	0.82305	0.2854
**Interaction**	-0.99499	6	-0.16583	-0.62036	0.4105
**Residual**	9.356	35	0.26731		
**Total**	10.19	46			
